# Simulated global warming affects endophytic bacterial and fungal communities of Antarctic pearlwort leaves and some bacterial isolates support plant growth at low temperatures

**DOI:** 10.1038/s41598-022-23582-2

**Published:** 2022-11-06

**Authors:** Michele Perazzolli, Bianca Vicelli, Livio Antonielli, Claudia M. O. Longa, Elisa Bozza, Laura Bertini, Carla Caruso, Ilaria Pertot

**Affiliations:** 1 Centre Agriculture, Food and the Environment (C3A), University of Trento, Via E. Mach 1, 38098 San Michele all’Adige, Italy; 2 Research and Innovation Centre, Fondazione Edmund Mach, Via E. Mach 1, 38098 San Michele all’Adige, Italy; 3 Center for Health and Bioresources, Bioresources Unit, AIT Austrian Institute of Technology GmbH, Konrad-Lorenz-Strasse 24, 3430 Tulln an der Donau, Austria; 4 Department of Ecological and Biological Sciences, University of Tuscia, Largo dell’Università s.n.c., 01100 Viterbo, Italy

**Keywords:** Microbiology, Molecular biology, Plant sciences, Environmental sciences

## Abstract

Antarctica is one of the most stressful environments for plant life and the Antarctic pearlwort (*Colobanthus quitensis*) is adapted to the hostile conditions. Plant-associated microorganisms can contribute to plant survival in cold environments, but scarce information is available on the taxonomic structure and functional roles of *C. quitensis*-associated microbial communities. This study aimed at evaluating the possible impacts of climate warming on the taxonomic structure of *C. quitensis* endophytes and at investigating the contribution of culturable bacterial endophytes to plant growth at low temperatures. The culture-independent analysis revealed changes in the taxonomic structure of bacterial and fungal communities according to plant growth conditions, such as the collection site and the presence of open-top chambers (OTCs), which can simulate global warming. Plants grown inside OTCs showed lower microbial richness and higher relative abundances of biomarker bacterial genera (*Allorhizobium-Neorhizobium-Pararhizobium-Rhizobium*, *Aeromicrobium*, *Aureimonas*, *Hymenobacter*, *Novosphingobium*, *Pedobacter*, *Pseudomonas* and *Sphingomonas*) and fungal genera (*Alternaria*, *Cistella,* and *Vishniacozyma*) compared to plants collected from open areas (OA), as a possible response to global warming simulated by OTCs. Culturable psychrotolerant bacteria of *C. quitensis* were able to endophytically colonize tomato seedlings and promote shoot growth at low temperatures, suggesting their potential contribution to plant tolerance to cold conditions.

## Introduction

Antarctica is the coldest region of the world, and it is one of the most limiting and stressful environments for plant life^1^. Only a small part of the Antarctic surface, corresponding to less than 1% of the continent^2^, is suitable for plant colonization and it is represented by the ice-free areas mainly found along the Antarctic Peninsula, associated islands, and coastal areas, also known as Maritime Antarctica^3^. In these areas, plant survival is limited by extreme environmental conditions, such as low temperature, desiccation, wind abrasion, high radiation, low water availability, and poor nutrient availability^3,4^. Only two angiosperms can grow in the Antarctic environments, namely *Colobanthus quitensis* and *Deschampsia antarctica*^3^. *Colobanthus quitensis*, known as the Antarctic pearlwort, is a small-sized cushion-like perennial herb, and it is the only dicotyledonous plant described in the Antarctic environment^5^. The Antarctic ecotype of *C. quitensis* possesses complex physiological mechanisms, allowing its survival under hostile environmental conditions^4^, such as high photosynthetic performance under low temperatures^6^ and a good tolerance to freeze–thaw cycles, UV-B radiation, low water availability, and osmotic stress^5^. In addition to plant mechanisms, plant-associated microorganisms can also contribute to plant tolerance to abiotic stresses and improve physiological acclimation^7^.

Plants are associated with a wide range of microorganisms, which may establish mutualistic interactions^8^. In particular, the leaf phyllosphere is colonized by a large variety of bacterial and fungal communities with beneficial effects on plant growth and tolerance to biotic and abiotic stresses^9^. Some plant-associated microorganisms can obtain nutrition and shelter from the plant, and, in exchange, they can promote plant growth and survival under stressful conditions by producing phytohormones and enhancing plant nutrient uptake^8^. Microorganisms living inside plant tissues are known as plant endophytes and they can establish long-lasting interactions with their host with precise regulations of the primary and secondary metabolism^10,11^. For example, bacterial endophytes can increase plant tolerance to cold stress and improve plant photosynthetic activity, carbohydrate content, cell osmotic regulations, nutrient acquisition, auxin biosynthesis, ethylene regulation, antioxidant content, and membrane fluidity under low temperatures^12,13^. *Colobanthus quitensis*-associated communities have been partially investigated, demonstrating that Actinobacteria, Bacteroidetes, Firmicutes, and Proteobacteria are the most abundant (dominant) bacterial phyla^14–17^, while Ascomycota and Basidiomycota are the dominant fungal phyla^5,18–20^. However, *C. quitensis*-associated microorganisms were mostly explored using culture-dependent approaches^5,14,15,18–23^ and the effect of plant growth conditions on the taxonomic structure of endophytic bacterial and fungal communities of *C. quitensis* is almost unknown. Moreover, the functional properties of *C. quitensis*-associated microorganisms were mainly reported for endophytic fungi^12,24–29^, while less information is available for endophytic bacteria.

Climate change is one of the main concerns nowadays and it is severely impacting Antarctic environments^30^ with consequent melting of perennial snow and increased colonization by plants^31^. Moreover, global warming can affect the taxonomic structure of phyllosphere microbial communities^32^. Passive warming systems, such as open-top chambers (OTCs), have been proposed as a reasonable approach to simulate global warming for remote areas, such as polar habitats^33–35^, because they can increase the midday temperature of about 4°C^31^. Although the use of passive warming systems (i.e. OTC) is controversial^36^, OTCs have been applied to evaluate plant physiological responses in Antarctic habitats, such as photosynthetic parameters^34^, freezing resistance^37^, metatranscriptome^35^ and proteome^31^ of *C. quitensis*. To the best of our knowledge, OTCs were never used to study the possible impact of increased temperatures on the taxonomic structure of *C. quitensis*-associated microorganisms. The aim of this study was to assess the possible impacts of simulated global warming on plant endophytes in Antarctic environments, by evaluating the effect of OTCs on the taxonomic structure of endophytic bacterial and fungal communities of *C. quitensis* leaves using culture-independent analyses. In addition, we isolated and characterized some psychrotolerant bacterial endophytes of *C. quitensis* leaves to understand their contribution to plant growth at low temperatures.

## Results

### Composition of endophytic bacterial and fungal communities of *Colobanthus quitensis* leaves

*Colobanthus quitensis* plants were collected from open areas (OA samples) of three Antarctic sites (S1, close to the beach, near penguin colonies; S2, 300 m from the coast, 20 masl; S3, 550 m from the coast, 30 masl) and inside open-top chambers (OTC samples) that were available in two sites (S2 and S3; Supplementary Fig. 1). A total of 664 bacterial (782,480 filtered read counts) and 96 fungal (2,600,410 filtered read counts) amplicon sequence variants (ASVs) were obtained (Supplementary Table 1, 2, and 3). Of the bacterial and fungal ASVs, 83% and 82% were assigned to taxa at the family level; 62% and 68% to taxa at the genus level, respectively (Supplementary Tables 1 and 2). Bacterial communities were dominated by Actinobacteria, Bacteroidetes, Deinococcus-Thermus, Patescibacteria, and Proteobacteria phyla, and by *Flavobacterium*, *Massilia*, *Pedobacter*, *Pseudomonas*, and *Sphingomonas* genera in terms of relative abundances (Supplementary Fig. 2a and Supplementary Table 1). Moreover, fungal communities were dominated by Leotiomycetes (Ascomycota) and Tremellomycetes (Basidiomycota) classes, and by *Tetracladium* and *Vishniacozyma* genera (Supplementary Fig. 2b and Supplementary Table 2).

Richness (expressed as the number of observed ASVs) of bacterial and fungal communities (Supplementary Table 3) differed according to the presence of OTCs (*P* ≤ 0.05) and to the collection site (*P* ≤ 0.05) based on generalized linear models (GLMs; Supplementary Table 4). In particular, bacterial and fungal richness was lower in OTC samples compared to OA samples (*P* ≤ 0.05), according to the estimated marginal mean comparisons (Supplementary Table 4). Moreover, bacterial richness was higher in S2 compared to S3, while fungal richness was higher in S2 and S3 compared to S1. The alpha-diversity (estimated with the Simpson’s index) of bacterial and fungal communities was affected by the collection site (*P* ≤ 0.05, GLMs of OA samples collected from S1, S2, and S3), but not by the presence of OTCs (*P* > 0.05), with higher bacterial alpha-diversity in S2 compared to S3 and higher fungal alpha-diversity in S2 and S3 compared to S1, according to the estimated marginal mean comparisons (Supplementary Table 4).

### The open-top chamber and collection site affect the taxonomic structure of endophytic bacterial and fungal communities of *Colobanthus quitensis* leaves

**Figure 1:**
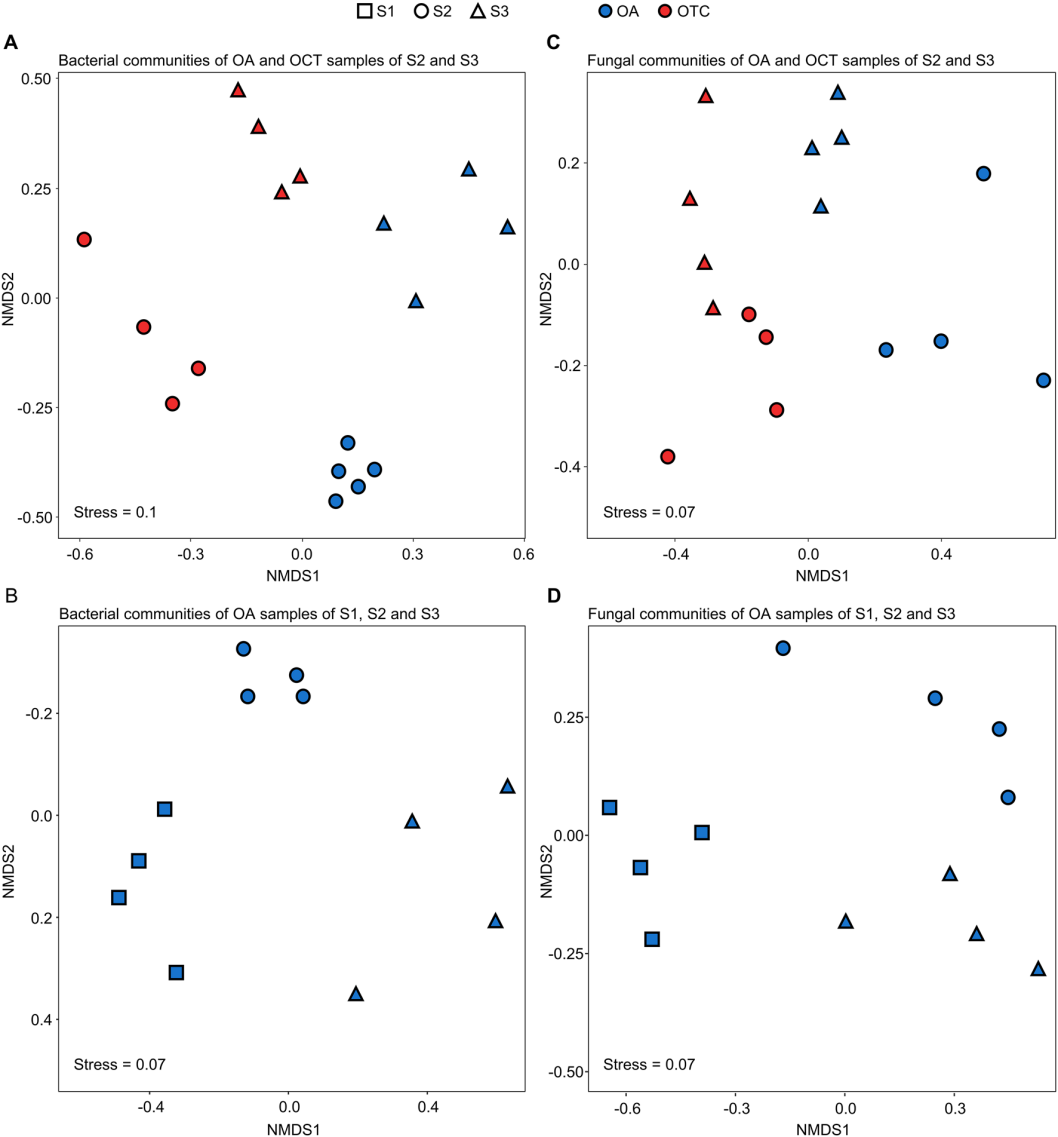
Non-metric multidimensional scaling (NMDS) endophytic bacterial (**A**,**B**) and fungal (**C**,**D**) communities of *Colobanthus quitensis*. NMDS plots are reported for amplicon sequence variants (ASVs) obtained from *C. quitensis* leaves collected in open areas (OA samples; blue) of the Antarctic site 1 (S1; square), site 2 (S2; circle), and site 3 (S3; triangle) or inside open-top chambers (OTC samples; blue) that were available in two sites (S2 and S3). Since OTCs were not available in S1, two datasets were analyzed: OA and OTC samples collected from S2 and S3 (**A**,**C**) or OA samples collected from S1, S2, and S3 (**B**,**D**). NMDS stress values are reported on each panel. Significant effects of the presence of OTCs and the collection site were found according to the permutational multivariate analysis of variance (Supplementary Table 5).

A non-metric multidimensional scaling (NMDS) plot was obtained on Bray–Curtis dissimilarity distances of beta-diversity analysis on the dataset of OA and OTC samples collected from the S2 and S3 sites (first dataset), and it discriminated bacterial communities according to the presence of OTCs and the collection site on the first and second axis, respectively (Fig. 1A). Likewise, the NMDS plot discriminated bacterial communities according to the collection site, when OA samples collected from the S1, S2, and S3 sites were considered as a second dataset for the analysis (Fig. 1B). Fungal communities clustered according to the presence of OTCs on the first axis and they were not clearly discriminated among the collection sites in the NMDS plot of OA and OTC samples collected from S2 and S3 (Fig. 1C). However, the effect of the collection site on fungal communities was better highlighted in the NMDS plot of OA samples collected from S1, S2, and S3 (Fig. 1D).

The permutational multivariate analysis of variances (PERMANOVA) on Bray–Curtis dissimilarities confirmed significant differences in bacterial communities according to the presence of OTCs (*P* = 0.001, pseudo F = 3.70, R2 = 0,18 and Df = 1) and to the collection site (*P* = 0.001, pseudo F = 2.87, R2 = 0.14 and Df = 1) in the dataset of OA and OTC samples collected from S2 and S3, with a contribution of 14.1% and 18.3% on the beta-diversity, respectively (Supplementary Table 5a). The collection site affected (*P* = 0.002, pseudo F = 3.34, R2 = 0.43 and Df = 2) bacterial communities (contribution of 42.6% on the beta-diversity) when OA samples collected from S1, S2, and S3 were analyzed as second dataset (Supplementary Table 5b). In agreement with the NMDS plot, fungal communities differed according to the presence of OTCs (contribution of 47.8% on the beta-diversity) in the dataset of OA and OTC samples collected from S2 and S3 (*P* = 0.001, pseudo F = 14.8, R2 = 0.48 and Df = 1; Supplementary Table 5c), and according to the collection site (contribution of 56.6% on the beta-diversity) in the dataset of OA samples collected from S1, S2 and S3 (*P* = 0.015 , pseudo F = 5.87, R2 = 0.57 and Df = 2; Supplementary Table 5d).

**Figure 2:**
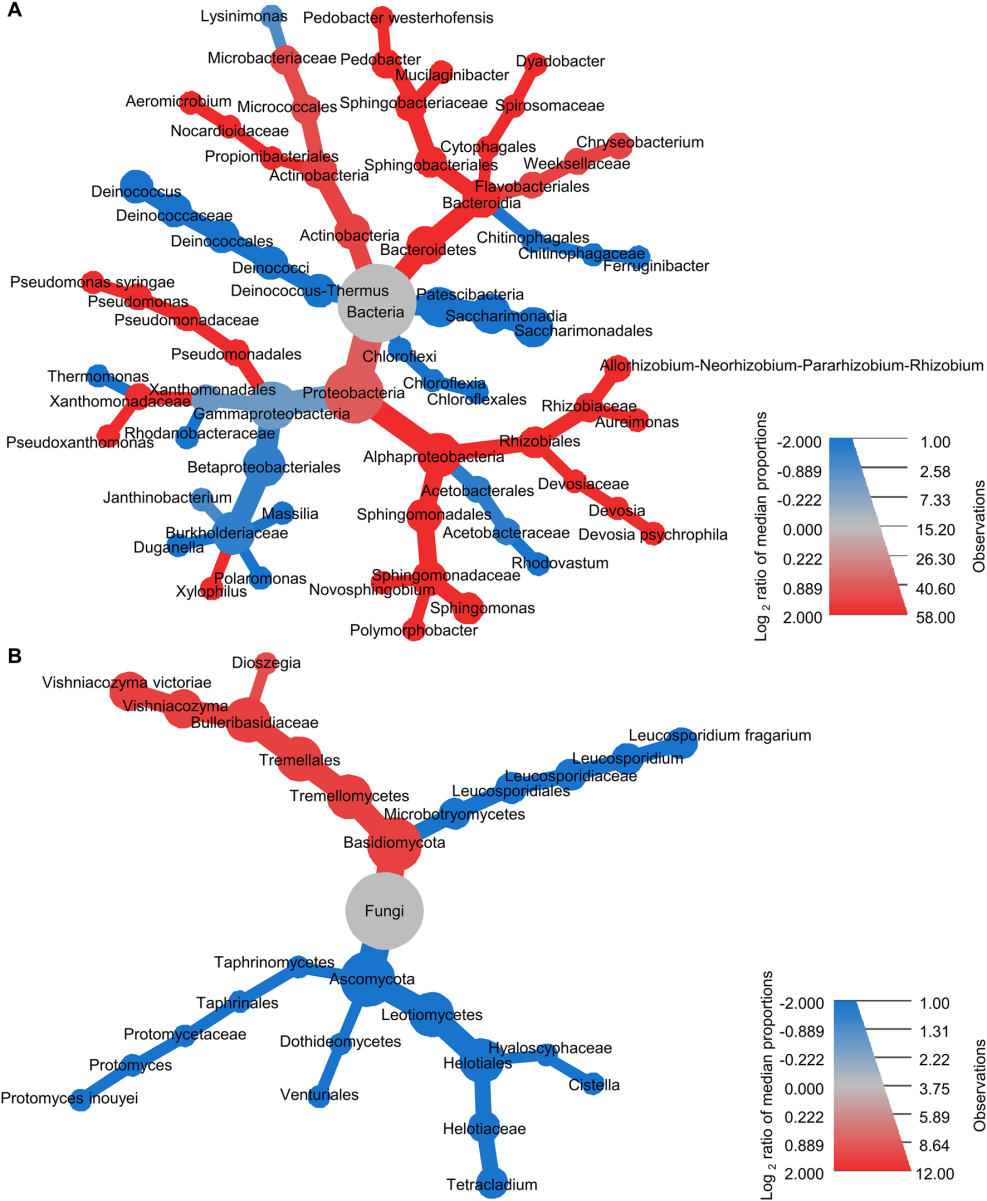
Heat tree of endophytic bacterial (**A**) and fungal (**B**) taxa of *Colobanthus quitensis* affected by the presence of open-top chambers (OTCs). Amplicon sequence variants (ASVs) affected by the presence of OTCs were identified by indicator taxon analysis with Random Forest models (Supplementary Tables 6 and 8) on *C. quitensis* leaves collected in open areas (OA samples) or inside open-top chambers (OTC samples) of the Antarctic site 2 (S2) and site 3 (S3). A non-parametric Wilcoxon Rank Sum test was applied to highlight differences in the relative abundances of each taxon in OTC samples compared to OA samples. Taxa with increased and decreased relative abundances in OTC and OA samples are shown respectively in red and blue, according to the colored scale legend of Log_2_-transformed median proportion. For each taxon, the dimension of the node is proportional to the total number of read counts.

**Figure 3:**
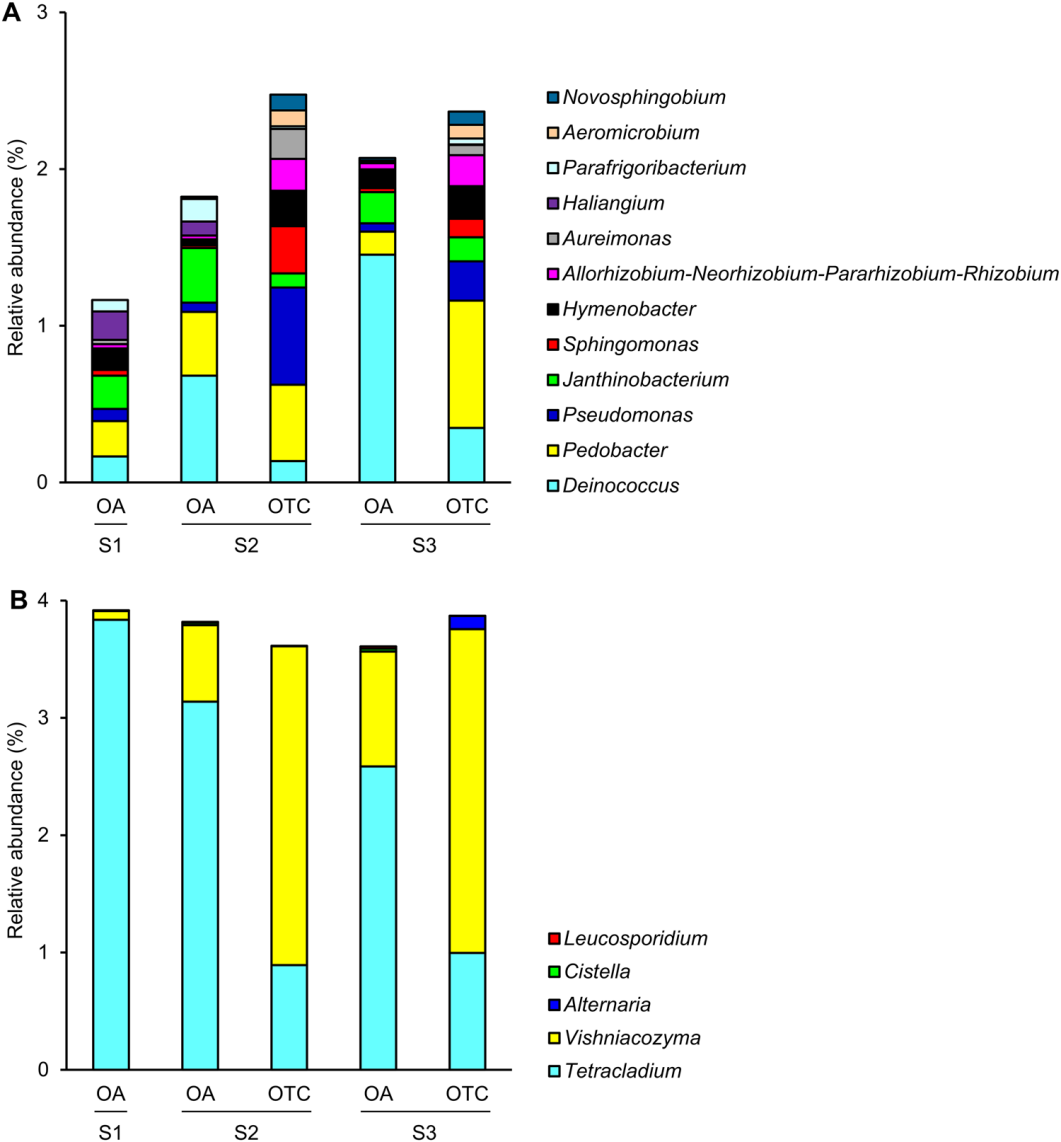
Histograms of endophytic bacterial (**A**) and fungal (**B**) taxa of *Colobanthus quitensis* affected by the presence of open-top chambers (OTCs). Amplicon sequence variants (ASVs) affected by the presence of OTCs and by the collection site were identified by indicator taxon analysis with Random Forest models, followed by a permutational test (Supplementary Tables 7 and 9). Relative abundances (%) of ASVs affected by the presence of OTCs were then calculated at the level of genus for samples of *C. quitensis* leaves collected in open areas (OA samples) or inside open-top chambers (OTC samples) of the Antarctic site 1 (S1), site 2 (S2) and site 3 (S3).

Differences in bacterial community structure between the OA and OTC samples were associated with 62 ASVs that belonged mostly to *Acetobacteraceae* (3 ASVs), *Burkholderiaceae* (13 ASVs), *Deinococcaceae* (5 ASVs), *Microbacteriaceae* (3 ASVs), *Pseudomonadaceae* (3 ASVs), *Sphingobacteriaceae* (7 ASVs) and *Sphingomonadaceae* (4 ASVs) families, according to the indicator taxon analysis with Random Forest models on the first dataset (OA and OTC samples collected from S2, and S3; Supplementary Table 6A). In particular, Actinobacteria, Alphaproteobacteria, and Bacteroidia were more represented in OTC samples, while Chloroflexia, Deinococcus-Thermus, and Gammaproteobacteria were more represented in OA samples, according to the Wilcoxon Rank Sum test applied on the relative abundances in OTC and OA samples (Fig. 2A). The permutational test analysis on ASVs selected by the indicator taxon analysis revealed higher relative abundances of *Allorhizobium-Neorhizobium-Pararhizobium-Rhizobium*, *Aeromicrobium*, *Aureimonas*, *Hymenobacter*, *Novosphingobium*, *Pedobacter*, *Pseudomonas* and *Sphingomonas* in OTC samples compared to OA samples, and higher relative abundances of *Deinococcus*, *Haliangium*, *Janthinobacterium,* and *Parafrigoribacterium* in OA samples compared to OTC samples (Fig. 3A, Supplementary Tables 7a and 7b). The indicator taxon analysis with Random Forest models on the second dataset (OA samples collected from S1, S2, and S3) revelated that 75 ASVs contributed to the differences in bacterial community structure among the collection sites, and they belonged mostly to *Burkholderiaceae* (9 ASVs), *Deinococcaceae* (4 ASVs), *Microbacteriaceae* (6 ASVs), *Sphingobacteriaceae* (10 ASVs), *Sphingomonadaceae* (4 ASVs), *Spirosomaceae* (7 ASVs), *Weeksellaceae* (4 ASVs) families (Supplementary Table 6B). In particular, the relative abundances of *Chryseobacterium*, *Mucilaginibacter,* and *Rhodococcus* were higher in S1 compared to the other two sites, those of *Galbitalea*, *Parafrigoribacterium* and *Pedobacter* were higher in S2, and those of *Deinococcus*, *Devosia* and *Hymenobacter* were higher in S3, according to the permutational analysis of variances (permutational ANOVA) analysis (Supplementary Fig. 3a and Supplementary Table 7c).

In fungal communities, 15 ASVs (belonging mostly to *Bulleribasidiaceae*, *Helotiaceae,* and *Leucosporidiaceae* families) mainly contributed to the differences between the OA and OTC samples, according to the indicator taxon analysis with Random Forest models (Supplementary Table 8a). Basidiomycota (Tremellomycetes) and Ascomycota (Dothideomycetes, Leotiomycetes, and Taphrinomycetes) were more represented in OTC samples and OA samples, respectively, according to the Wilcoxon Rank Sum test on the relative abundances (Fig. 2B). In particular, higher relative abundances of *Alternaria*, *Cistella,* and *Vishniacozyma* were found in OTC samples compared to OA samples, while higher relative abundances of *Leucosporidium* and *Tetracladium* were found in OA samples compared to OTC samples, according to the permutational test analysis (Fig. 3B, Supplementary Tables 9a and 9b). Moreover, 22 ASVs mainly contributed to the differences in fungal communities among the three collection sites, and they belonged mostly to *Bulleribasidiaceae* (6 ASVs), *Helotiaceae* (2 ASVs), *Helotiales* (6 ASVs), and *Hyaloscyphaceae* (2 ASVs) families (Supplementary Table 8b). In particular, the relative abundances of *Lachnum* were higher in S2 compared to the other two sites, those of *Bullera*, *Vishniacozyma*, and *Dioszegia* were higher in S3, while those of *Tetracladium* were higher in S1 and S2 compared to S3, according to the permutational ANOVA analysis (Supplementary Fig. 3b and Supplementary Table 9c).

### Culturable endophytic bacteria of *Colobanthus quitensis* leaves promote plant growth under cold conditions

Counts of culturable endophytic bacteria of *C. quitensis* leaves were comparable among the Antarctic samples and incubation temperatures (15 ± 1 °C and 25 ± 1 °C; Supplementary Fig. 4). According to morphological analysis of microbial colonies, 79 endophytic bacterial isolates were selected for taxonomic annotation, and they belonged to *Duganella* (3.8%), *Ewingella* (1.3%), *Flavobacterium* (1.3%), *Hafnia* (11.4%), *Janthinobacterium* (2.5%), *Pedobacter* (3.8%), *Pseudomonas* (59.5%), *Rahnella* (7.6%), *Rhodanobacter* (1.3%), Serratia (1.3%), *Sphingomonas* (3.8%) and *Yersinia* (2.5%) genera (Supplementary Table 10).

**Figure 4:**
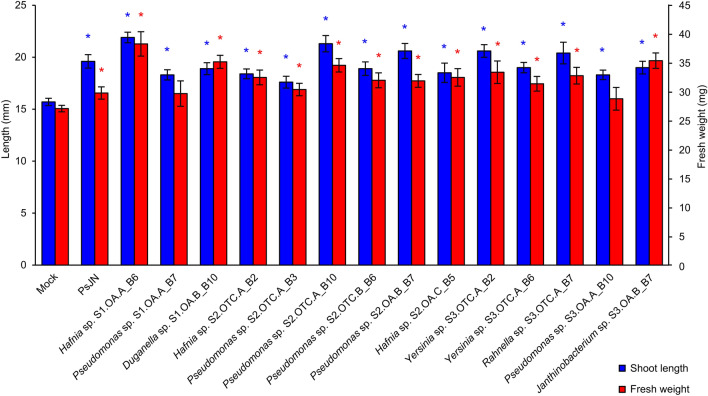
Growth promotion of tomato seedlings by psychrotolerant bacterial isolates of *Colobanthus quitensis*. The shoot length (mm, blue bars) and fresh weight (mg/plant; red bars) were analyzed for mock-inoculated plants (Mock) and plants inoculated with psychrotolerant bacterial isolates obtained from *C. quitensis* leaves. Assessments were carried out 15 days after seed inoculation and plant incubation at 15 ± 1 °C. *Paraburkholderia phytofirmans* PsJN (PsJN) was used as a positive control for its ability to promote plant growth at chilling temperatures^72^. Mean and standard error values of 10 replicates (dishes with five plants each) from two experiments are presented for each treatment. For each parameter, colored asterisks indicate significant growth promotion in the pairwise comparisons between mock-inoculated and bacterium-inoculated plants, according to the Mann–Whitney test (*P* ≤ 0.05). No differences in root length were found.

To limit redundancies, 38 representative endophytic bacterial isolates were selected according to taxonomic annotation and 14 of them grew well at low temperatures (4 ± 1 °C, 10 ± 1 °C, and 15 ± 1 °C), such as six *Pseudomonas* sp., four *Hafnia* sp., one *Duganella* sp., one *Janthinobacterium* sp., one *Rahnella* sp. and one *Yersinia* sp. isolates (Supplementary Table 10). All these psychrotolerant *C. quitensis* bacterial isolates endophytically colonized tomato seedlings (Supplementary Fig. 5) and promoted tomato shoot length (Fig. 4) at 15 days after seed inoculation and plant incubation at 15 ± 1 °C. Moreover, plant fresh weight was promoted by seed inoculation with *Duganella* sp. S1.OA.B_B10, *Hafnia* sp. S1.OA.A_B6, *Hafnia* sp. S2.OA.C_B5, *Hafnia sp*. S2.OTC.A_B2, *Janthinobacterium* sp. S3.OA.B_B7, *Pseudomonas sp*. S2.OA.B_B7, *Pseudomonas* sp. S2.OTC.A_B3, *Pseudomonas sp*. S2.OTC.A_B10, *Pseudomonas sp*. S2.OTC.B_B6, *Rahnella* sp. S3.OTC.A_B7, *Yersinia* sp. S3.OTC.A_B2, and *Yersinia* sp. S3.OTC.A_B6, (Fig. 4).

## Discussion

Plants growing under extreme environmental conditions, such as those of Antarctica, harbor complex microbial communities that may play key functional roles in plant growth and survival in cold environments^8^. Besides the agreement with previous findings on the taxonomic composition of bacterial and fungal communities associated with *C. quitensis* under cold conditions, our study highlighted taxonomic changes of endophytic bacterial and fungal communities according to the collection site and the simulated global warming obtained by OTCs. Moreover, the psychrotolerant bacterial endophytes tested were isolated from *C. quitensis* leaves collected from different growth conditions and showed comparable growth-promoting effects on tomato seedlings, suggesting a possible selection of beneficial bacteria by *C. quitensis*, regardless of the plant growth conditions.

Our amplicon sequencing analysis showed that endophytic bacterial communities of *C. quitensis* leaves were dominated by *Burkholderiaceae*, *Microbacteriaceae*, *Pseudomonadaceae*, *Sphingobacteriaceae,* and *Sphingomonadaceae* families. Moreover, the isolation of culturable endophytic bacteria of *C. quitensis* confirmed the dominance of *Burkholderiaceae* (*Duganella* sp.), *Hafniaceae* (*Hafnia* sp.), *Oxalobacteraceae* (*Janthinobacterium* sp.), *Pseudomonadaceae* (*Pseudomonas* sp.), *Sphingobacteriaceae* (*Pedobacter* sp.), *Sphingomonadaceae* (*Sphingomonas* sp.) and *Yersiniaceae* (*Rahnella* sp.). These findings agree with previous studies that demonstrated the colonization of *C. quitensis* endosphere and rhizosphere mainly by Actinobacteria, Bacteroidetes, Firmicutes, and Proteobacteria^16,23^. In particular, *Microbacteriaceae*, *Pseudomonadaceae,* and *Sphigomonadaceae* families are known to dominate *C. quitensis* endosphere and phyllosphere^17^, suggesting possible adaptation of endemic bacterial taxa to Antarctic conditions. For example, bacterial isolates of the rhizosphere of Antarctic plants displayed high metabolic plasticity^15^, as a possible adaptation strategy to the harsh environmental conditions. In addition, endophytic fungal communities were dominated by *Helotiaceae* (mainly *Tetracladium*; Ascomycota) and *Bulleribasidiaceae* (mainly *Vishniacozyma* and *Dioszegia*; Basidiomycota) families. Ascomycota and Basidiomycota are known to be the most abundant phyla of culturable fungi isolated from *C. quitensis* samples under Antarctic conditions, such as *Alternaria* sp., *Leucosporidium* sp., *Microdochium* sp., *Penicillium* sp., and *Vishniacozyma* sp.^5,18–20,38^.

The taxonomic structure of endophytic bacterial and fungal communities of *C. quitensis* leaves differed according to the collection site, which affected the parameters of richness, alpha-diversity, and beta-diversity. In particular, 75 bacterial ASVs and 22 fungal ASVs resulted as possible biomarkers of the collection site, and they belonged mostly to seven bacterial families (*Burkholderiaceae*, *Deinococcaceae*, *Microbacteriaceae*, *Sphingobacteriaceae*, *Sphingomonadaceae*, *Spirosomaceae,* and *Weeksellaceae*) and four fungal families (*Bulleribasidiaceae*, *Helotiaceae*, *Helotiales,* and *Hyaloscyphaceae*). In particular, *Chryseobacterium*, *Mucilaginibacter,* and *Rhodococcus* were possible biomarkers of the site close to the beach and near penguin colonies (S1), *Galbitalea*, *Parafrigoribacterium,* and *Pedobacter* resulted as possible biomarkers of the site located at 300 m from the coast (20 masl; S2), and *Deinococcus*, *Devosia,* and *Hymenobacter* resulted as possible biomarkers of the site located at around 550 m from the coast (30 masl; S3). Fungal biomarkers of the collection sites were *Lachnum* (for S2), *Bullera*, *Vishniacozyma,* and *Dioszegia* (for S3), and *Tetracladium* (for S1 and S2), suggesting that further studies are required to better understand the possible contribution of microclimatic conditions (e.g., temperature, humidity, wind, and snow), soil properties and the presence of plant or penguin species in shaping endophytic bacterial fungal communities of *C. quitensis* in the three collections sites. Although endophytic communities were analyzed in our study, migrations of some bacterial and fungal taxa from soil, wind, and animals could be possible^39^. For example, bacterial communities of *C. quitensis* rhizosphere can be influenced by neighboring plants (e.g., *D. antarctica*) and bird presence at the collection site^16^. Moreover, the relative abundances of *Micrococcaceae* and *Xanthomonadaceae* families of *C. quitensis* rhizosphere and endosphere can be affected by the geochemical soil background of the collection sites in Antarctic environments^23^.

In addition to the effect of the collection site, the presence OTCs severely affected the taxonomic structure of endophytic bacterial fungal communities of *C. quitensis* leaves. Alpha-diversity was not affected by the presence of OTCs, but the bacterial and fungal richness was lower in OTC samples compared to OA samples, as a possible limitation of some taxa under the simulated global warming. In particular, 62 bacterial ASVs and 15 fungal ASVs mainly contributed to the differences between OTC and OA communities, and these possible biomarkers of simulated global warming belonged mainly to seven bacterial families (*Acetobacteraceae*, *Burkholderiaceae*, *Deinococcaceae*, *Microbacteriaceae*, *Pseudomonadaceae*, *Sphingobacteriaceae* and *Sphingomonadaceae*) and three fungal families (*Bulleribasidiaceae*, *Helotiaceae,* and *Leucosporidiaceae*). For example, higher (e.g., *Allorhizobium-Neorhizobium-Pararhizobium-Rhizobium*, *Aeromicrobium*, *Aureimonas*, *Hymenobacter*, *Novosphingobium*, *Pedobacter*, *Pseudomonas* and *Sphingomonas*) and lower (e.g., *Deinococcus*, *Haliangium*, *Janthinobacterium*, and *Parafrigoribacterium*) relative abundances of bacterial genera were found in OTC samples compared to OA samples. Similarly, higher (e.g., *Alternaria*, *Cistella,* and *Vishniacozyma*) and lower (*Leucosporidium* and *Tetracladium*) relative abundances of fungal genera were found in OTC samples compared to OA samples. In Antarctic soils, the presence of OTCs has been reported to reduce the abundance of Gram-positive bacteria^40^, increase the ratio between Alphaproteobacteria and Acidobacteria^41^ and increase the relative abundances of methanogenic archaea (*Thermoplasmata*) and heterotrophic fungi (Ascomycota)^42^. Although OTCs are commonly used to simulate the increase in air temperature caused by global warming^33–35^, they can partially modify the moisture, wind, and fluxes of air-borne microorganisms with possible effects on the composition of plant-associated communities. Moreover, increased temperatures can also stimulate *C. quitensis* growth and reproduction^34,43^ with consequent indirect effects on microbial communities. However, global warming simulated by OTCs may have negligible effects when compared to long-term experiments^44^, indicating that long-term experiments under natural conditions on progressive warming are needed, to validate the effects of temperature increase on the composition of microbial communities in Antarctic environments.

We found that 14 endophytic bacterial isolates (six isolates of *Pseudomonas* sp., three isolates of *Hafnia* sp. two isolates of *Yersinia* sp., and one isolate of *Duganella* sp., *Janthinobacterium* sp. and *Rahnella* sp.) grew well at low temperatures (4 ± 1 °C, 10 ± 1 °C, and 15 ± 1 °C) and they were able to colonize tomato seedlings (leaves, shoots, and roots) and promote tomato growth at 15 ± 1 °C, suggesting their possible contribution to plant tolerance to cold conditions. Likewise, fungal isolates (*Lenzites* sp. and *Trametes* sp.) obtained from *C. quitensis* plants can display broad extracellular enzymatic profiles, promote secondary root development, and increase fresh weight in Arabidopsis and tomato seedlings with inhibitory activities against phytopathogenic fungi^38^, corroborating the beneficial effects of *C. quitensis* endophytes. It has been reported that plant growth of *C. quitensis* can be promoted by Antarctic rhizobacteria (e.g., *Enterobacter* sp.) and endophytic fungi (e.g., *P. chrysogenum*) under salinity stress^22^ and simulated climate changes^45^. Tomato seedlings were used according to previous studies on plant growth promotion at chilling temperatures (15 °C) with soil-borne psychrotolerant bacteria^46^, since surface-disinfected seeds can provide an environment with no bacterial contaminants, as confirmed by our re-isolation data of mock-inoculated plants. Although the compatibility with other plant species should be further confirmed, the endophytic colonization of tomato seedlings suggests a broad-spectrum activity of endophytic bacterial isolates of *C. quitensis*. Likewise, germination, growth, and physiological performance of crop plants (e.g., blueberry, cayenne, lettuce, onion, tomato, and wheat) can be improved under abiotic stresses (e.g. low temperature, drought, or salinity) by plant-associated bacteria (e.g., *Arthrobacter* sp. and *Planococcus* sp.), endophytic fungi (e.g., *Penicillium bialowienzense, P. brevicompactum*, *P. chrysogenum,* and *P. rubens*) or soil bacteria (e.g., *Pseudomonas* spp.) isolated from Antarctic environments^21,24,47,48^, indicating possible biotechnological applications of Antarctic isolates. For example, beneficial Antarctic bacteria (e.g. *Pseudomonas* sp., *Serratia* sp., and *Staphylococcus* sp.) and fungi (e.g., *Penicillium* sp., *Alternaria* sp., and *Geomyces* sp.) can modify hormonal content (e.g., abscisic acid, ethylene, indole-3-acetate, jasmonic acid, and salicylic acid)^14,49^, nutrient availability^25^ and gene expression in colonized plants^26,50^, indicating that further characterizations on bacterial properties and plant responses are required to better understand the mechanisms of cold tolerance activated by psychrotolerant Antarctic bacteria in crop plants.

In conclusion, the taxonomic structure of endophytic bacterial and fungal communities of *C. quitensis* leaves was shaped by the collection site and the simulation of global warming obtained by OTCs, suggesting a partial selection of bacterial and fungal taxa according to the environmental conditions. In particular simulated global warming increased (bacterial genera: *Allorhizobium-Neorhizobium-Pararhizobium-Rhizobium*, *Aeromicrobium*, *Aureimonas*, *Hymenobacter*, *Novosphingobium*, *Pedobacter*, *Pseudomonas* and *Sphingomonas*; fungal genera: *Alternaria*, *Cistella,* and *Vishniacozyma*) and decreased (bacterial genera: *Deinococcus*, *Haliangium*, *Janthinobacterium,* and *Parafrigoribacterium*; fungal genera: *Leucosporidium* and *Tetracladium*) the relative abundances of biomarker genera. Moreover, all psychrotolerant bacterial isolates tested (e.g., *Duganella* sp., *Hafnia* sp., *Janthinobacterium* sp., *Pseudomonas* sp., *Rahnella* sp., and *Yersinia* sp.) were isolated from *C. quitensis* plants collected from different growth conditions and showed comparable growth-promoting effects on tomato seedlings, suggesting a possible selection of beneficial bacteria by *C. quitensis*, regardless of the plant growth conditions. Although these results need to be further validated with data from other sites, possibly with shotgun metagenomic approaches and long-term measurements under natural conditions, they suggest that global warming could impact plant-associated microbial communities in Antarctic environments.

## Materials and methods

### Sample collection

*Colobanthus quitensis* samples were collected at King George Island near the Henryk Arctowski Polish Antarctic Station, Maritime Antarctica (62°14’ S, 58°48’ W) during the summer season (February 2018). Samples were collected inside the Antarctic Specially Protected Area (ASPA) 128 using permits provided by The Chilean Antarctic Institute (INACH) and by the Italian National Agency for New Technologies, Energy and Sustainable Economic Development-Technical Antarctic Unit (ENEA-UTA). In particular, *C. quitensis* plants were collected from open areas (OA samples) in three sites possibly differing in soil composition, altitude, and temperature: Site 1 (S1; 62°9′44.58″ S, 58°27′58.68″ W) located close to the beach, near penguin colonies; Site 2 (S2; 62°9′49.62″ S, 58°28′7.02″ W) located at around 300 m from the coast, at an altitude of 20 masl; Site 3 (S3; 62°9′52.90″ S, 58°28′ 21.31″ W) located at around 550 m from the coast, at an altitude of 30 masl. In two sites (S2 and S3), hexagonal transparent plexiglass OTCs were installed in December 2013, to evaluate the effects of warm temperatures on freezing tolerance and physiological processes of *C. quitensis* under field conditions^37^. OTCs were similar to those used in the International Tundra Experiment and they were made with transparent Plexiglass walls of 40 cm in height, punched with 25 holes of 1.5 cm diameter each to allow some wind to pass through and hence avoid an excessive increase in air temperature^37^. OTCs determined an increase of about 4 °C at midday and they have been proposed to simulate global warming for remote areas, such as polar habitats^33–35,37^. Thus, plant samples were collected inside OTCs (OTC samples) of the S2 and S3 sites, to assess the effect of simulated global warming on plant-associated communities.

For each condition, four replicates of *C. quitensis* leaves were collected randomly (pool of five plants), soaked in sterile RNAlater solution (Thermo Fisher Scientific), and stored at 4 °C. Plant samples were first moved to Chile and then to Italy maintaining the cold chain. Samples were processed within a few days after collection, upon arrival in Italy. *Colobanthus quitensis* leaves were surface-disinfected as described previously^51^. Briefly, leaves were treated with 70% ethanol for 1 min, 2% sodium hypochlorite for 1.5 min, and 70% ethanol for 1 min, followed by three washes with sterilized distilled water (2 min each), to analyze endophytic microorganisms of long-lasting and well-established plant–microbe interactions^10^, and to limit the characterization of epiphytic microorganisms possibly migrated by wind dispersal on *C. quitensis* leaves.

### DNA extraction, amplification, and sequencing of endophytic bacterial and fungal communities

For the culture-independent analysis, genomic DNA was extracted from surface-disinfected *C. quitensis* leaves (0.1 g) using the Nucleospin Plant II kit (Macherey–Nagel). The bacterial V5-V7 region of the 16S ribosomal DNA (rDNA) and fungal internal transcribed spacer 2 (ITS2) were amplified from 5 ng template DNA with a nested PCR approach, which is used to limit the amplification of host DNA in amplicon sequencing studies of plant endophytes^52–56^. The first bacterial 16S amplification was carried out with the primer 799 forward (5’-AACMGGATTAGATACCCKG-3’) and 1392 reverse (5’-ACGGGCGGTGTGTRC-3’), to exclude chloroplast 16S rDNA and to amplify bacterial and mitochondrial rRNA of 600 bp and 1,000 bp amplicon size, respectively^52^. Bacterial 16S amplicons (600 bp) were purified by agarose gel separation, followed by the NucleoSpin Gel Clean-up purification kit (Macherey–Nagel). The second 16S amplification was carried out using the purified product (2 µL) with the primer 799 forward (5’-AACMGGATTAGATACCCKG-3’) and 1175 reverse (5’-ACGTCRTCCCCDCCTTCCT-3’) that included Illumina adapters for library construction (5’-TCGTCGGCAGCGTCAGATGTGTATAAGAGACAG-3’ and 5’-GTCTCGTGGGCTCGGAGATGTGTATAAGAGACAG-3’, respectively). Bacterial 16S amplifications were obtained using the FastStart High-Fidelity PCR system (Roche) as described previously^57^ with 25 cycles (in the first 16S amplification) or 30 cycles (in the second 16S amplification) of amplification (95 °C for 30 s, 52 °C for 30 s and 72 °C for 30 s).

The first fungal ITS amplification was carried out with the primer ITS1 forward (5’-CTTGGTCATTTAGAGGAAGTAA-3’) and TW13 reverse (5’-GGTCCGTGTTTCAAGACG-3’), which amplifies fungal ITS and part of the ribosomal large subunit^58^. The second PCR amplification was adapted from Tedersoo et al.^59^ using the product of the first amplification (3 µl) with equimolar mixes of the ITS3Mix forward primers (5’-CATCGATGAAGAACGCAG-3’, 5’-CAACGATGAAGAACGCAG-3’, 5’-CACCGATGAAGAACGCAG-3’, 5’-CATCGATGAAGAACGTAG-3’ and 5’-CATCGATGAAGAACGTGG-3’)^60^ and the ITS4Mix reverse primers (5’-TCCTCCGCTTATTGATATGC-3’ and 5’-TCCTSSSCTTATTGATATGC-3’), to increase coverage of the fungal kingdom^53^. All primers included the Illumina adapters (5’-TCGTCGGCAGCGTCAGATGTGTATAAGAGACAG-3’ and 5’-GTCTCGTGGGCTCGGAGATGTGTATAAGAGACAG-3’ in the forward and reverse primers, respectively). Fungal ITS amplifications were obtained using the FastStart High-Fidelity PCR system (Roche) as described previously^57^ with 30 cycles of amplification in the first and second ITS amplification (95 °C for 30 s, 60 °C for 30 s, and 72 °C for 30 s).

All reactions were carried out in duplicate and pooled after amplification. DNA indexing, quantification, and library preparation for the Illumina MiSeq sequencing (PE300) were carried out as described previously^57^. Sequences of the 40 samples [three locations (S1, S2, and S3), two conditions for S2 and S3 (OA and OTC samples), two amplicons (bacterial 16S and fungal ITS), and four replicates] were obtained (PRJNA644506).

### Sequence processing and bioinformatic analysis

Illumina reads were filtered with Bowtie2 v2.4.2^61^, and sequence quality was checked with FastQC v0.11.9 (https://www.bioinformatics.babraham.ac.uk/projects/fastqc/). Primers were cut using Cutadapt v3.4 (https://cutadapt.readthedocs.io/en/v3.4/). Sequences were quality filtered, trimmed, denoised and amplicon sequence variants (ASVs) were generated with DADA2 v1.18.0^62^. Denoised forward and reverse ASV sequences were merged, and chimeras were removed. Filtered bacterial ASVs were checked using Metaxa2 v2.2.3^63^ for targeting the presence of the hypervariable regions of the 16S rRNA gene, from V5 to V7. Filtered fungal ASVs were screened using ITSx v1.1.3^64^ for targeting the ITS2 region. Taxonomic assignment of 16S rRNA gene and ITS2 ASVs was performed using the RDP classifier implemented in DADA2 against the SILVA v138.1^65^ and UNITE 8.3 database^66^, respectively. A bacterial and fungal table of read counts was built and imported into the R-4.1.2 statistical environment for further analyses (https://www.r-project.org/). After taxonomic classification, ASVs classified as plastid rRNA, and other than archaea, bacteria, or fungi were removed.

### Statistical analysis

Since OTCs were not available on the S1 site, two datasets were generated for statistical analysis of bacterial and fungal communities. The first dataset included OA and OTC samples collected from S2 and S3 and it was used to assess the effects of the collection site and the presence of OTCs. To analyze the effect of the collection site in all sites, only OA samples collected from S1, S2 and S3 were considered as a second dataset for the analyses.

In each dataset, low abundant ASVs of bacterial and fungal data were filtered out as possible aleatory taxa or contaminants, and only ASVs with a maximum relative abundance greater than 0.1% in at least one sample were kept to find solid differences between samples^67^. Richness (observed ASVs) and alpha-diversity (Simpson’s index) values were calculated by averaging the results after multiple rarefactions (999 iterations), using the rtk R package^68^. For each dataset, GLMs were generated on richness and alpha-diversity values, assuming a Gamma distribution of the data. The models used as predictors were the collection site (i.e. differences between S2 and S3 sites) and the presence of OTCs (i.e. differences between OTC and OA samples) in the first dataset (OA and OTC samples collected from S2 and S3), or the collection site only (i.e. differences between S1, S2, and S3 sites) in the second dataset (OA samples collected from S1, S2 and S3). The GLMs were inspected with diagnostic residual plots followed by the analysis of deviance with Chi-squared statistics. A post-hoc analysis with pairwise multiple comparisons (estimated marginal mean comparisons) was conducted using the emmeans R package (https://cran.r-project.org/web/packages/emmeans/index.html).

Multiple rarefactions were applied to each dataset of bacterial and fungal ASVs (minimum sequence depth of 24,976 and 84,453 reads, respectively), and results of 999 iterations were averaged to account for differences in sequencing depth^57^. Exploratory unsupervised analysis of beta diversity patterns was performed with NMDS on Bray–Curtis dissimilarities, followed by PERMANOVA. Legitimation of PERMANOVA’s assumptions was verified by the analysis of multivariate homogeneity of group dispersions. NMDS, PERMANOVA, and beta-dispersion analysis were performed using the vegan R package (https://CRAN.R-project.org/package=vegan), while NMDS plots were generated using the ggvegan (https://gavinsimpson.github.io/ggvegan/) and ggplot2 (https://CRAN.R-project.org/package=ggplot2) R packages.

For the indicator taxon analysis, rarefied count data were processed with the mikropml R package (https://CRAN.R-project.org/package=mikropml), variables (ASVs) with zero or near-zero variance were removed and correlated features were collapsed. For each dataset, pre-processed data were then used to train Random Forest models, with the collection site and the OTC factor as an outcome. Model performances were evaluated with repeated k-fold cross-validation (tenfold, 10 repetitions) and parameters were tuned by choosing mtry values between 1 and the square root of the total number of variables. Model training was accomplished with the caret R package (https://topepo.github.io/caret/), mtry values that determined the highest model accuracy were chosen and input to Random Forest analysis. Variable importance was assessed with permutations (999 iterations), using the rfPermute R package (https://CRAN.R-project.org/package=rfPermute). ASVs with significant mean decrease accuracy (*P* ≤ 0.05) were extracted (selected ASVs) and used to generate heat trees of bacterial and fungal taxa using the Metacoder R package (https://CRAN.R-project.org/package=metacoder) to highlight differences in the relative abundances between OTC and OA samples, according to a non-parametric Wilcoxon Rank Sum test (*P* ≤ 0.05). Differential abundance analysis was conducted on selected ASVs with a non-parametric permutational test and permutational ANOVA with 999 iterations implemented in the RVAideMemoire R package (https://CRAN.R-project.org/package=RVAideMemoire), in order to assess in which growing condition (pairwise comparisons between OTC and OA samples) and in which collection site (multiple comparisons of OA samples collected from S1, S2 and S3) selected ASVs were enriched, respectively. ASVs with a relative abundance significantly different (*P* ≤ 0.05) either between OTC or OA samples or between the collection sites were extracted, parsed at the genus level, and used to generate barplots with the RAM R package (https://CRAN.R-project.org/package=RAM) and ternary plots with the ggtern R package (https://cran.r-project.org/web/packages/ggtern/index.html).

### Isolation of culturable endophytic bacteria of *Colobanthus quitensis* leaves

For the culture-dependent analysis, surface-disinfected leaves (0.5 g) were ground in stainless jars using a mixer-mill disruptor (MM 400) at 25 Hz for 2 min in the presence of 1.0 mL NaCl 0.85%. Culturable bacteria were isolated by plating serial dilutions of each *C. quitensis* suspension (100 µL aliquots) on Antarctic Bacterial Medium (ABM; 5 g/L peptone and 2 g/L yeast extract; Oxoid) supplemented with 100 mg/L cycloheximide. As control of plant surface disinfection, the last washing solution (10 mL) was centrifuged (3,500 g for 10 min) and the pellet was plated to confirm the absence of bacterial growth. Plates were incubated at 15 ± 1 °C and 25 ± 1 °C and bacterial colony forming units (CFU) per unit of plant fresh weight (CFU/g) were assessed daily for up to 60 days^5^. Three replicates (0.5 g) were analyzed for each Antarctic site and condition (S1.OA, S2.OA, S2.OTC, S3.OA, and S3.OTC) and two technical replicates were analyzed for each sample.

Representative endophytic bacterial isolates of *C. quitensis* were selected for each sample based on morphological visual observation of bacterial colonies (namely size, color, opacity, texture, form, elevation, and margin) grown in NA, as described previously^69,70^ and they were further selected according to the taxonomic annotation to avoid redundancy. For taxonomic annotation, the bacterial V6-V8 16S region of *C. quitensis* bacterial isolates was amplified by colony PCR with the DreamTaq DNA Polymerase (ThermoFisherScientific) using specific primer pairs (27f.: 5’-AGAGTTTGATCCTGGCTCAG-3’ and 1492r: 5’-GGTTACCTTGTTACGACTT-3’ for bacterial 16S). PCR products were purified by the NucleoSpin PCR Clean-up purification kit (Macherey–Nagel) and sequenced with an ABIPRISM 3730xl DNA analyzer (ThermoFisherScientific). Sanger sequences were deposited in the NCBI database and taxonomic annotation was carried out using by BLAST search against the NCBI nucleotide database (E-value 1 × 10^−5^, max target 100, max HSPS 5) and BLAST hits were processed with BlobTools v.1.1.1, parsing the information according to the best sum of bit score criterion.

### Assessment of bacterial growth at low temperatures

Each representative endophytic bacterial isolate of *C. quitensis* was grown overnight (18 h) in ABM at 25 ± 1 °C at 80 rpm^71^. Bacterial cells were collected by centrifugation (3,500 g for 10 min), washed three times with sterile 10 mM MgSO_4_, and resuspended in sterile 10 mM MgSO_4_ to adjust bacterial suspension to 0.01 optical density at 600 nm (OD_600_). Plates (diameter of 120 mm) containing solid ABM (ABM and 15 g/L technical agar, Oxoid) were inoculated with 10 µL drops of each bacterial suspension and they were incubated at 4 ± 1 °C, 10 ± 1 °C, 15 ± 1 °C and 25 ± 1 °C for 48 h, 96 h and 120 h. Bacterial growth was assessed visually for each bacterial isolate at each temperature and time point, and it was scored in classes as follows: 0, no growth; 1, very limited growth; 2, limited growth; 3, growth equivalent to *Paraburkholderia phytofirmans* PsJN, which was used as reference endophytic strain of plant growth promotion at chilling temperatures^72^; 4, abundant growth; 5 very abundant growth. To select psychrotolerant bacterial isolates, three replicates (spotted colonies) were analyzed for each bacterial isolate and the experiment was carried out twice.

### Tomato seed inoculation and growth conditions

Each psychrotolerant bacterial isolate was grown overnight (18 h) in ABM at 15 ± 1 °C at 80 rpm and bacterial cells were collected by centrifugation (3,500 g for 10 min) and washing (three times) with sterile 10 mM MgSO_4_, as described above. The bacterial suspension was adjusted to 1.0 × 10^8^ CFU/mL based on the OD_600_ conversion table optimized for each isolate (Supplementary Table 10).

Seeds of *Solanum lycopersicum* L. cultivar Moneymaker (Justseed) were treated with 70% ethanol for 1 min, 2% sodium hypochlorite for 5 min, and 70% ethanol for 1 min, followed by three washes with sterilized distilled water (3 min each) in a 50 mL-tube with moderate shaking, to reduce the number of seed-associated microorganisms as described previously^51^. Surface-disinfected seeds (100 seeds for each treatment) were treated with 5 mL of sterile 10 mM MgSO_4_ (mock-inoculated) or inoculated with 5 mL of the bacterial suspension (bacterium-inoculated) of a psychrotolerant bacterial isolate (1.0 × 10^8^ CFU/mL) by overnight (18 h) incubation at 15 ± 1 °C in a sterile 15 mL-tube under orbital shaking at 80 rpm. As a control, seeds were inoculated with *P. phytofirmans* PsJN, because this strain promotes tomato growth^51^ and improves grapevine growth at chilling temperatures^72^. Seeds of each inoculation were transferred to 100 cm^2^-square dishes (Sarstedt; 100 seeds for each dish) containing 8 g/L water agar (Oxoid) and they were incubated for four days in a growth chamber (Bertagnin) at 15 ± 1 °C with a 16 h photoperiod (photon flux density of 0.050 mmol/sec/m^2^) to allow seed germination.

Germinated seeds with the same root length (2 mm) were selected and five seeds were transferred along a line at 4 cm from the upper edge of a 100 cm^2^-square dish containing 40 mL solid (8 g/L agar) half-strength Hoagland. Dishes were incubated in a vertical position in the growth chamber (15 ± 1 °C with a 16 h photoperiod) to assess plant growth promotion under chilling temperature^46^. Shoot length and root length were measured with a ruler and the fresh weight of the whole plant was assessed with a precision balance at 15 days after seed inoculation. Five replicates (dishes with five plants each) were analyzed for each treatment and the experiment was carried out twice.

### Bacterial re-isolation from tomato plants

At the end of the incubation period (15 days after seed inoculation), mock-inoculated and bacterium-inoculated plants (roots, shoot, and leaves) were collected, and each replicate (pool of five plants of a square dish) was surface-disinfected in a 50 mL-tube with 70% ethanol for 1 min, 2% sodium hypochlorite for 1.5 min and 70% ethanol for 1 min as described previously^51^. Plants were ground in a stainless jar using a mixer-mill disruptor (MM 400, Retsch) at 25 Hz for 45 s in the presence of 500 µL sterile 10 mM MgSO_4_. Each suspension was serially diluted and plated in triplicates on Luria Bertani agar (Oxoid). CFU values of endophytic bacterial strains were calculated per unit of plant fresh weight (CFU/g) two days after incubation at 15 ± 1 °C. Five replicates (dishes with five plants each) with two technical replicates (plates) were analyzed for each treatment and the experiment was carried out twice.

Tomato growth data and bacterial re-isolation data (Log_10_-transformed) were analyzed with Statistica 13.3 software (Tibco). Each experimental repetition was analyzed individually, and Kruskal–Wallis test was used to demonstrate non-significant differences between the two experiments (*P* > 0.05). Data from the two experimental repetitions were pooled and significant differences among treatments were assessed with the Mann–Whitney test (*P* ≤ 0.05) and the Kruskal–Wallis test (*P* ≤ 0.05) in the case of pairwise and multiple comparisons, respectively.

### Ethical approval

We confirm that all the experimental research and field studies on plants (either cultivated or wild), including the collection of plant material, complied with relevant institutional, national, and international guidelines and legislation. All of the material is owned by the authors and/or no permissions are required.

## Supplementary Information

Supplementary Information 1.

Supplementary Information 2.

## Data Availability

Amplicon sequencing data are available from NCBI SRA (PRJNA644506). Sanger sequencing accession numbers are available from NCBI Nucleotide (accession numbers reported in Supplementary Table 10).
