# Supplementary material for: Simulated global warming affects endophytic bacterial and fungal communities of Antarctic pearlwort leaves and some bacterial isolates support plant growth at low temperatures
Source: Sci Rep. 2022 Nov 6;12:18839. doi: 10.1038/s41598-022-23582-2 (PMC9637742; doi:10.1038/s41598-022-23582-2)
Supplement: Supplementary file 1 — Supplementary Information 1. [file 41598_2022_23582_MOESM1_ESM.pdf]

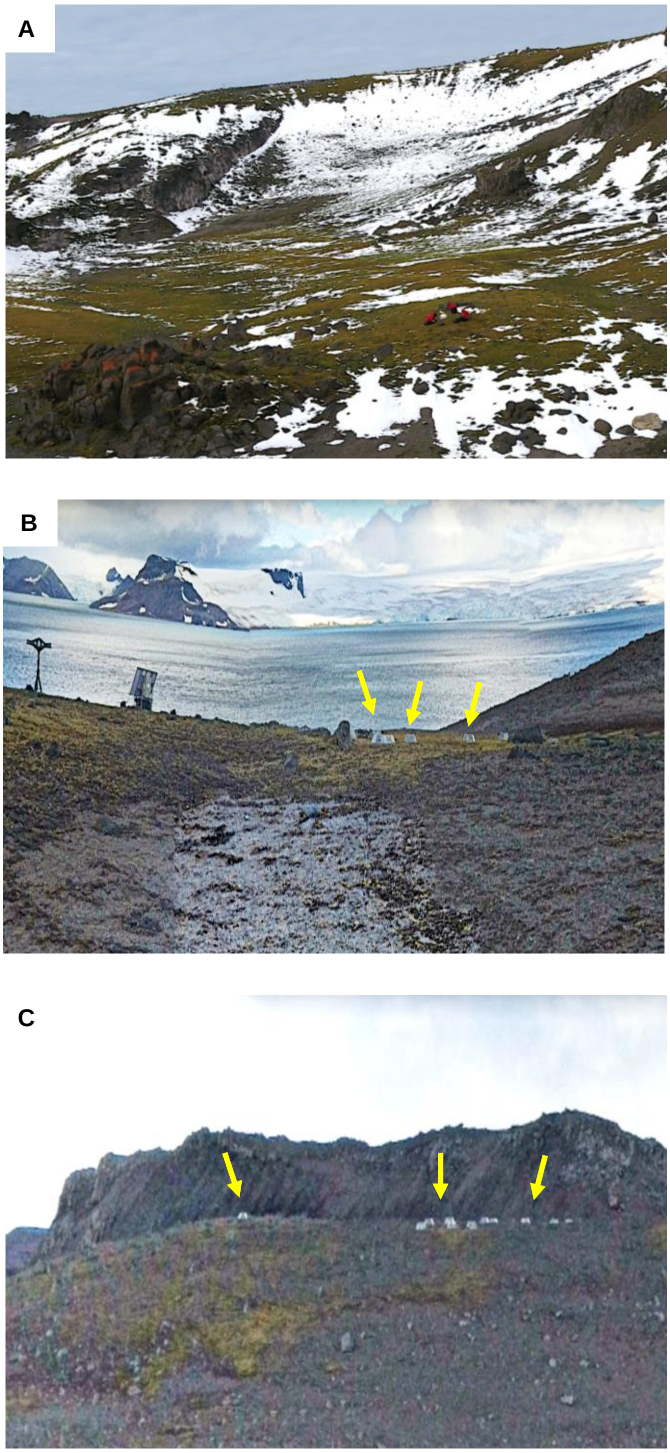

Supplementary Figure 1. Collection sites. *Colobanthus quitensis* leaf samples were collected at the King George Island of Antarctica near the Henryk Arctowski Polish Antarctic Station (https://arctowski.aq/en/about-station/) during the summer season (February 2018) from open areas (OA samples) in three sites differing in soil composition, altitude, and temperature: Site 1 (S1) located close to the beach, near penguin colonies (A); Site 2 (S2) located at around 300 m from the coast, at an altitude of 20 masl (B); Site 3 (S3) located at around 550 m from the coast, at an altitude of 30 masl (C). In two sites (S2 and S3), hexagonal transparent plexiglass open-top chambers (OTCs) were present, and plant samples were collected inside them (OTC samples).

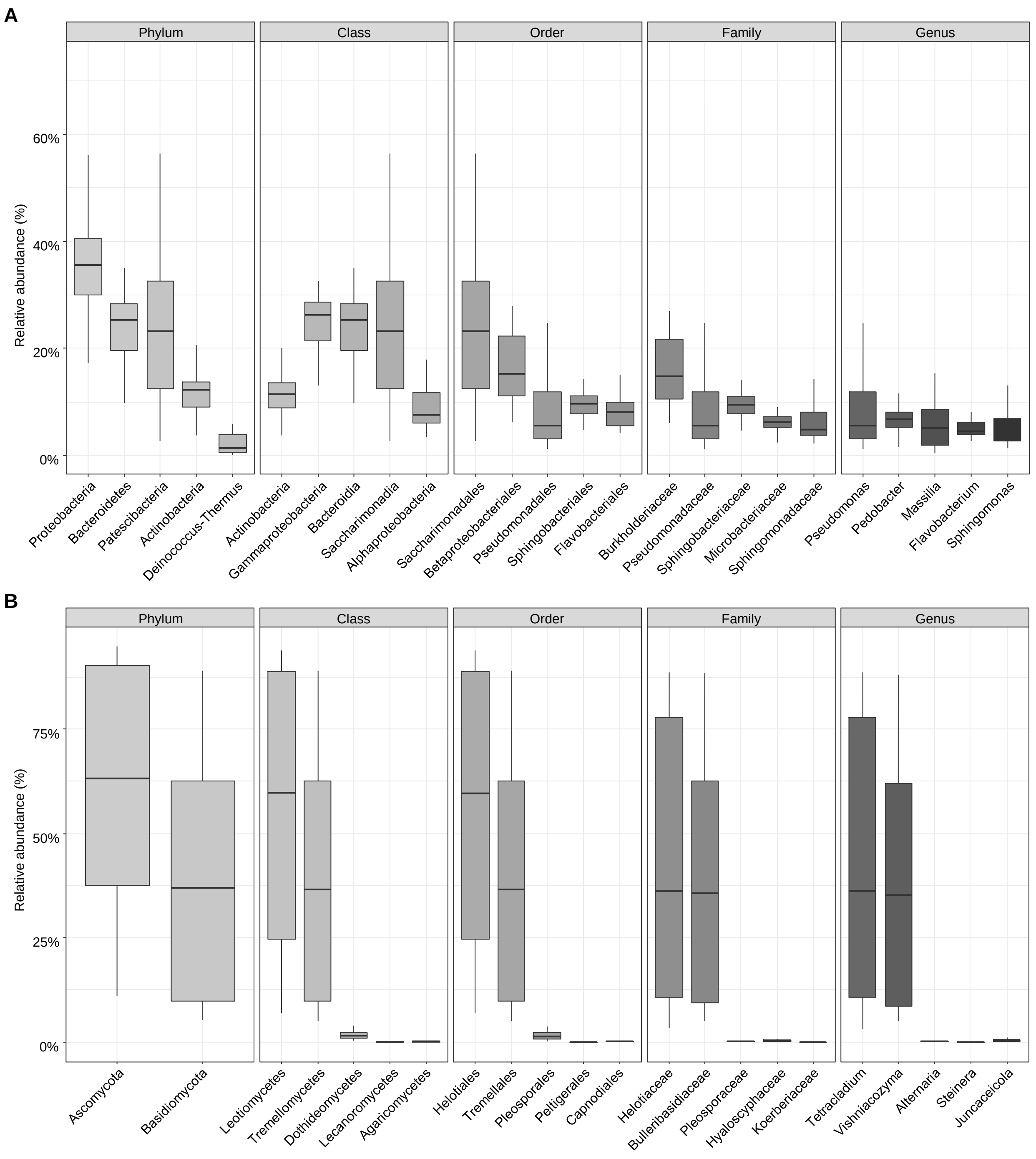

Supplementary Figure 2. Relative abundances of dominant endophytic microbial taxa of *Colobanthus quitensis* leaves. Box plots indicate the relative abundances of dominant bacterial (A) and fungal (B) taxa that were taxonomically annotated at the level of phylum, class, order, family, and genus from *C. quitensis* leaves collected in open areas (OA samples) of the Antarctic site 1 (S1), site 2 (S2) and site 3 (S3) or inside open-top chambers (OTC samples) that were available in two sites (S2 and S3).

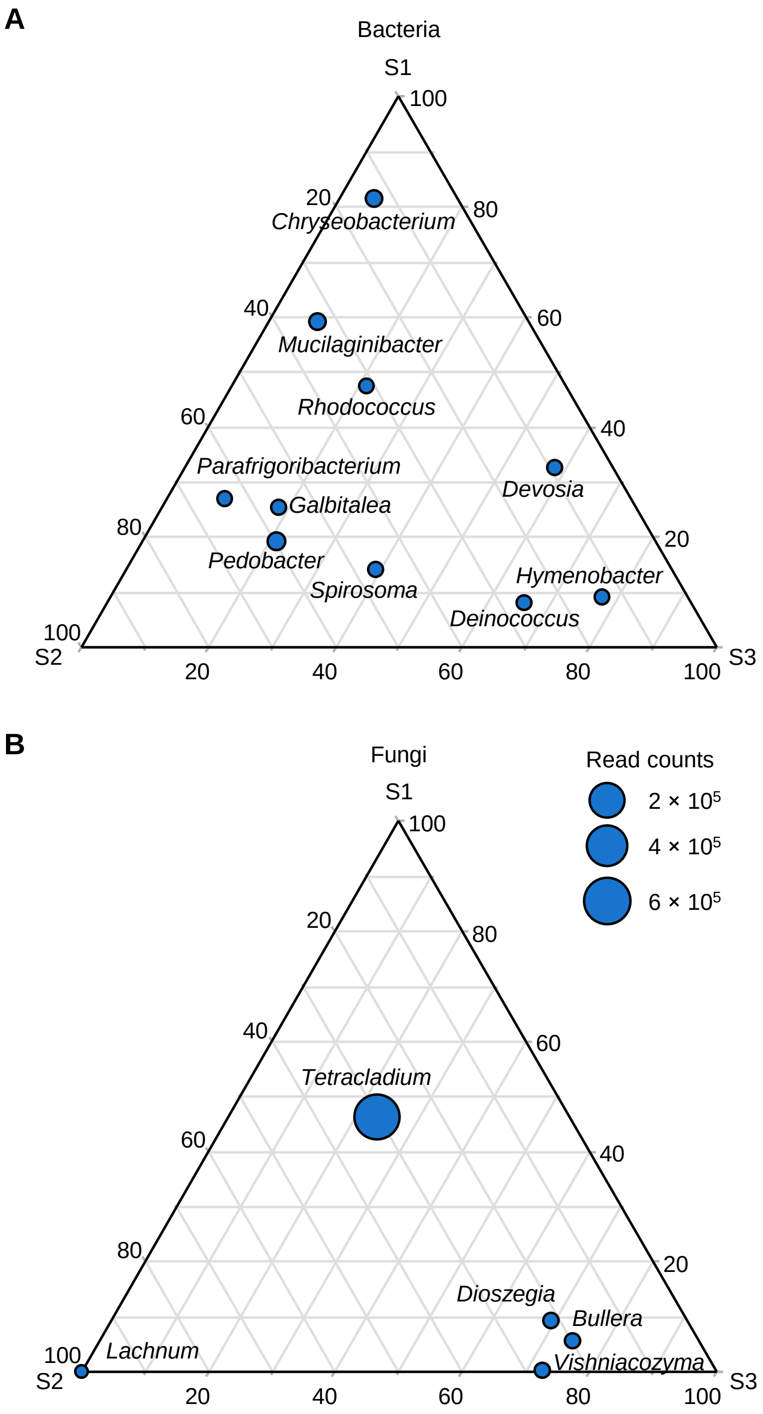

Supplementary Figure 3. Representation of endophytic bacterial (**a**) and fungal (**b**) taxa of *Colobanthus quitensis* affected by the collection site. Amplicon sequence variants (ASVs) affected by the collection site were identified by indicator taxon analysis with Random Forest models, followed by a permutational analysis of variance (Supplementary Tables 7 and 9), on *C. quitensis* leaves collected in open areas (OA samples) of the Antarctic site 1 (S1), site 2 (S2) and site 3 (S3). The circle dimension indicates the read counts of each taxon, according to the scale legend. The coordinates of each circle on each of the three axes are derived from the relative abundance of the respective genus in each of the three sites.

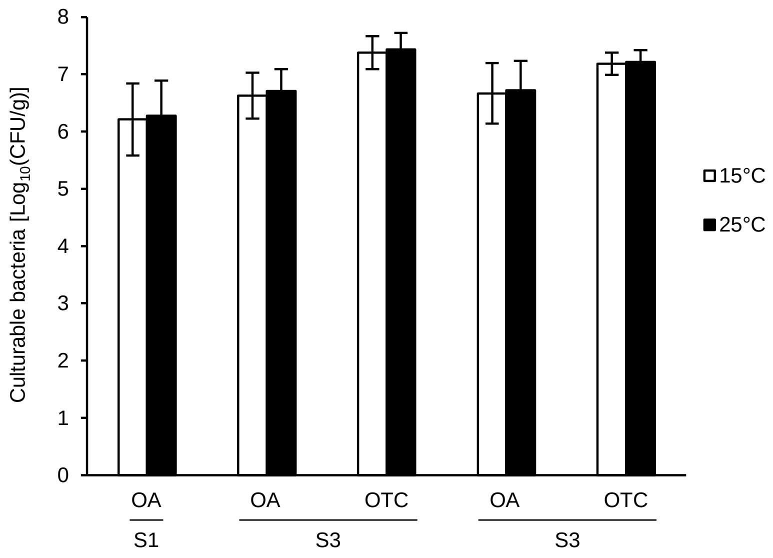

Supplementary Figure 4. Culturable endophytic bacteria of *Colobanthus quitensis* leaves. Colony forming units (CFU) of culturable endophytic bacteria per unit of leaf fresh weight (g) were assessed for *C. quitensis* leaves collected in open areas (OA samples) of the Antarctic site 1 (S1), site 2 (S2), and site 3 (S3) or inside open-top chambers (OTC samples) that were available in two sites (S2 and S3). Culturable bacteria were grown at 15 ± 1°C (white) and 25 ± 1°C (black) for 21 days on Nutrient Agar (NA) supplemented with 100 mg/l cycloheximide. No additional colonies were observed for a longer incubation time. Mean Log_{10} (CFU/g) and standard error values from three replicates are presented for each sample. No significant differences among conditions were found, according to the Kruskal–Wallis test ($P > 0.05$).

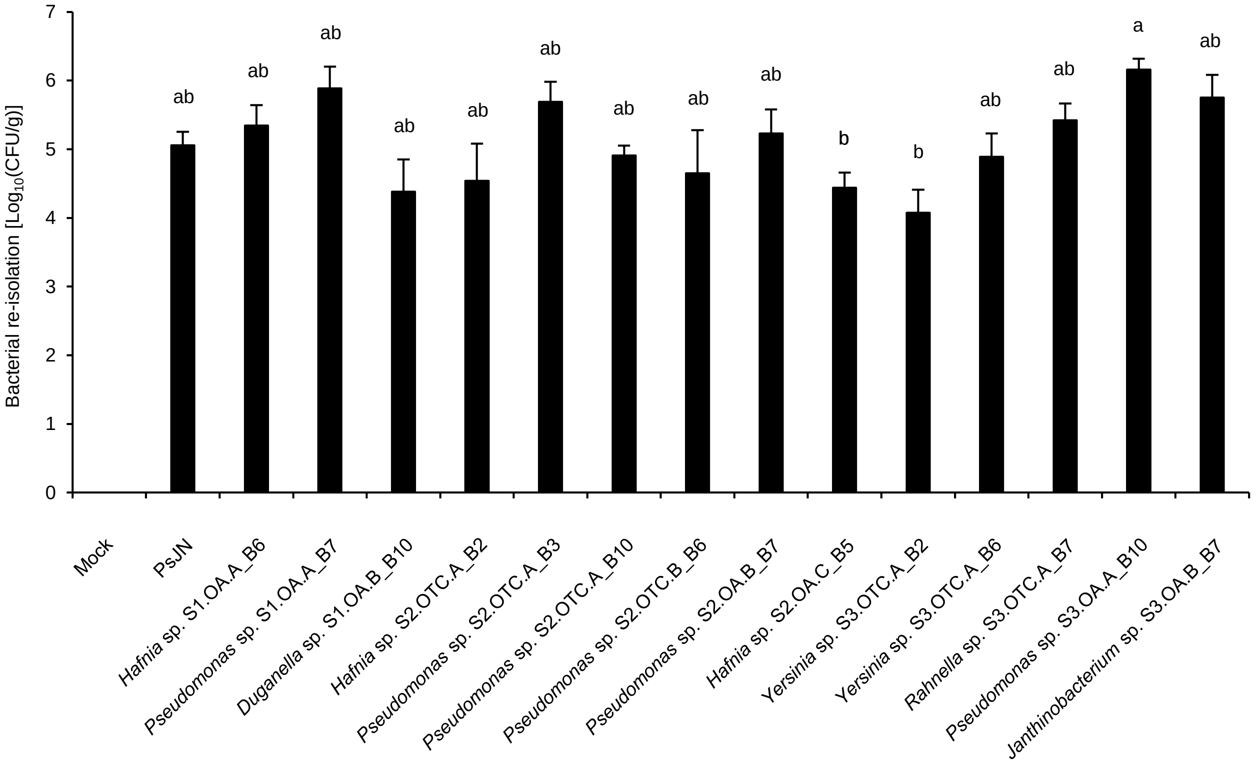

Supplementary Figure 5. Re-isolation of psychrotolerant *Colobanthus quitensis* bacterial isolates from tomato seedlings. The quantity of re-isolated bacteria was assessed for mock-inoculated plants (mock) and plants inoculated with psychrotolerant bacterial isolates (obtained from *C. quitensis* leaves) at 15 days after seed inoculation and plant incubation at 15 ± 1°C. *Paraburkholderia phytofirmans* PsJN (PsJN) was used as a positive control for its ability to colonize tomato plants. Bacterial counts were assessed two days after incubation on Luria Bertani agar at 15 ± 1°C and they were expressed as colony forming units per gram of fresh weight (CFU/g) of tomato plants (leaves, shoots, and roots). Mean and standard error values of five replicates (pool of five plants each) are reported for each treatment. Different letters indicate significant differences among inoculated plants according to the Kruskal–Wallis test ($P \leq 0.05$).

Supplementary tables (Excel file)

Supplementary Table 1. Amplicon sequence variants (ASVs) of endophytic bacterial communities of *Colobanthus quitensis* leaves.

Bacterial ASVs were identified in *C. quitensis* leaves collected in open areas (OA samples) of the Antarctic site 1 (S1), site 2 (S2), and site 3 (S3) or inside open-top chambers (OTC samples) that were available in two sites (S2 and S3). Taxonomic annotation (columns B-H), filtered read counts (columns I-AB) rarefied read counts (columns AC-BD), and relative abundances (columns BE-BX) are reported for each replicate (named from R1 to R4). Mean and standard error values of ASV relative abundances are calculated for each sample (columns BY-CH).

Supplementary Table 2. Amplicon sequence variants (ASVs) of endophytic fungal communities of *Colobanthus quitensis* leaves.

Fungal ASVs were identified in *C. quitensis* leaves collected in open areas (OA samples) of the Antarctic site 1 (S1), site 2 (S2), and site 3 (S3) or inside open-top chambers (OTC samples) that were available in two sites (S2 and S3). Taxonomic annotation (columns B-H), filtered read counts (columns I-AB) rarefied read counts (columns AC-BD), and relative abundances (columns BE-BX) are reported for each replicate (named from R1 to R4). Mean and standard error values of ASV relative abundances are calculated for each sample (columns BY-CH).

Supplementary Table 3. Summary of amplicon sequencing analysis of endophytic bacterial and fungal communities of *Colobanthus quitensis* leaves.

Filtered read counts, observed amplicon sequence variants (richness), and Simpson’s index (alpha-diversity) are reported for each replicate (named from R1 to R4) of endophytic bacterial and fungal communities obtained from *C. quitensis* leaves collected in open areas (OA samples) of the Antarctic site 1 (S1), site 2 (S2) and site 3 (S3) or inside open-top chambers (OTC samples) that were available in two sites (S2 and S3).

Supplementary Table 4. Factors affecting the richness and alpha-diversity of endophytic bacterial and fungal communities of *Colobanthus quitensis* leaves.

Generalized linear models (GLMs) were generated on richness (observed amplicon sequence variants) and alpha-diversity (Simpson's index) data of endophytic bacterial (A and B) and fungal (C and D) communities of *C. quitensis* leaves collected in open areas (OA samples) of the Antarctic site 1 (S1), site 2 (S2) and site 3 (S3) or inside open-top chambers (OTC samples) that were available in two sites (S2 and S3). Since OTCs were not available in S1, two datasets were analyzed: OA and OTC samples collected from S2 and S3 (first dataset) or OA samples collected from S1, S2, and S3 (second dataset), in order to assess the effects of the presence of OTCs and the collection site. A post-hoc analysis with estimated marginal mean (EMM) comparisons was carried out to better highlight differences between the OA and OTC samples ($P \leq 0.05$) or among collections sites ($P \leq 0.05$).

Supplementary Table 5. Factors affecting the beta-diversity of endophytic bacterial and fungal communities of *Colobanthus quitensis* leaves.

A permutational multivariate analysis of variance (PERMANOVA) was carried out using Bray-Curtis dissimilarities on endophytic bacterial (A and B) and fungal (B and C) communities of *C. quitensis* leaves collected in open areas (OA samples) of the Antarctic site 1 (S1), site 2 (S2) and site 3 (S3) or inside open-top chambers (OTC samples) that were available in two sites (S2 and S3), in order to assess effects of the presence of OTCs or the collection site. Since OTCs were not available in S1, two datasets were analyzed: OA and OTC samples collected from S2 and S3 (A and C) or OA samples collected from S1, S2, and S3 (B and D). The percentage (%) of the contribution of each factor to beta-diversity is reported in column E.

Supplementary Table 6. Amplicon sequence variants (ASVs) of endophytic bacteria of *Colobanthus quitensis* leaves affected by the presence of open-top chambers (A) and by the collection site (B).

Endophytic bacterial ASVs were identified in *C. quitensis* leaves collected in open areas (OA samples) of the Antarctic site 1 (S1), site 2 (S2), and site 3 (S3) or inside open-top chambers (OTC samples) that were available in two sites (S2 and S3). Indicator taxon analysis with Random Forest models highlighted bacterial ASVs that mainly contributed to the differences between OA and OTC samples collected from S2 and S3 (A; columns A-P) and among OA samples collected from S1, S2, and S3 (B; columns R-AI). ASVs are sorted according to a decreased accuracy of relevance (Mean Decrease Accuracy).

Supplementary Table 7. Differential abundance of bacterial amplicon sequence variants (ASVs) affected by the presence of open-top chambers (A, B) and by the collection site (C).

Endophytic bacterial ASVs were identified in *C. quitensis* leaves collected in open areas (OA samples) of the Antarctic site 1 (S1), site 2 (S2), and site 3 (S3) or inside open-top chambers (OTC samples) that were available in two sites (S2 and S3). Permutational analysis was carried out on ASVs selected by indicator taxon analysis with Random Forest models (Supplementary Table 6), in order to identify significant changes in abundances ($P \leq 0.05$) between OA and OTC samples (permutational t-test) of S2 (A; columns A-Q) and S3 (B; columns T-AJ), or among the three collection sites (permutational ANOVA) for OA samples (C; columns AM-BD). ASVs with significant changes in abundance are reported in bold.

Supplementary Table 8. Amplicon sequence variants (ASVs) of endophytic fungi of *Colobanthus quitensis* leaves affected by the presence of open-top chambers (A) and by the collection site (B).

Endophytic fungal ASVs were identified in *C. quitensis* leaves collected in open areas (OA samples) of the Antarctic site 1 (S1), site 2 (S2), and site 3 (S3) or inside open-top chambers (OTC samples) that were available in two sites (S2 and S3). Indicator taxon analysis with Random Forest

models highlighted fungal ASVs that mainly contributed to the differences between OA and OTC samples collected from S2 and S3 (A; columns A-P) and among OA samples collected from S1, S2, and S3 (B; columns R-AI). ASVs are sorted according to a decreased accuracy of relevance (Mean Decrease Accuracy).

Supplementary Table 9. Differential abundance of fungal amplicon sequence variants (ASVs) affected by the presence of open-top chambers (A, B) and by the collection site (C).
Endophytic fungal ASVs were identified in *C. quitensis* leaves collected in open areas (OA samples) of the Antarctic site 1 (S1), site 2 (S2), and site 3 (S3) or inside open-top chambers (OTC samples) that were available in two sites (S2 and S3). Permutational analysis was carried out on ASVs selected by indicator taxon analysis with Random Forest models (Supplementary Table 8), to identify significant ($P \leq 0.05$) changes in abundances between OA and OTC samples (permutational t-test) of S2 (A; columns A-Q) and S3 (B; columns T-AJ), or among the three collection sites (permutational ANOVA) for OA samples (C; columns AM-BD). ASVs with significant changes in abundance are reported in bold.

Supplementary Table 10. Endophytic bacterial isolates of *Colobanthus quitensis* leaves.
Endophytic bacterial isolates were selected according to colony morphology for each replicate (named A, B, and C) of *C. quitensis* leaves collected in open areas (OA samples) of the Antarctic site 1 (S1), site 2 (S2), and site 3 (S3) or inside open-top chambers (OTC samples) that were available in two sites (S2 and S3). Isolates were taxonomically annotated by the amplification of the bacterial 16S region and sequences are available at the NCBI database (http://www.ncbi.nlm.nih.gov/sra) under accession numbers reported for each bacterial isolate (Column D).
The growth of representative endophytic bacterial isolates of *C. quitensis* bacterial was assessed visually at 48, 96 and 120 h after incubation at 4 ± 1°C, 10 ± 1°C, 15 ± 1°C, and 25 ± 1°C on a solid Antarctic bacterial medium (Columns E-P) and scored as follows: 0, no growth; 1, very limited

growth; 2, limited growth; 3, growth equivalent to *Paraburkholderia phytofirmans* PsJN, which was used as reference endophytic strain of plant growth promotion at chilling temperatures (Barka et al. 2006 Appl. Environ. Microbiol. 72, 7246-7252); 4, abundant growth; 5 very abundant growth. Mean scores of six replicates (spotted colonies) from two experiments are reported for each isolate and time point.

Psychrotolerant *C. quitensis* bacterial isolates that grew well at low temperatures are reported in bold and their conversion of optical density (OD600 = 0.1) and colony forming units (CFU/mL) is reported in Column Q.
